# The Effects of Co-Culturing ND7/23 Sensory Neuron-like Cells and IFRS1 Schwann Cells on Myelination: A Single-Arm Nonrandomized Study

**DOI:** 10.3390/neurolint17090138

**Published:** 2025-09-01

**Authors:** Shizuka Takaku, Kazunori Sango

**Affiliations:** Diabetic Neuropathy Project, Department of Diseases and Infection, Tokyo Metropolitan Institute of Medical Science, Setagaya-ku, Tokyo 156-8506, Japan; takaku-sz@igakuken.or.jp

**Keywords:** sensory neuron-like cells, immortalized Schwann cells, co-culture, myelination, mitomycin C

## Abstract

**Background/Objectives**: Co-culture models of neurons and Schwann cells have been used to explore the mechanisms of myelination during development, axonal regeneration after injury, and the pathogenesis of various demyelinating neuropathies. A spontaneously immortalized Fischer rat Schwann cell line 1 (IFRS1), established from the primary culture of adult Fischer344 rat peripheral nerves, can myelinate neurites in co-cultures with primary cultured dorsal root ganglion neurons and neuronal cell lines, such as nerve growth factor (NGF)-primed PC12 cells and NSC-34 motor neuron-like cells. In this study, we aimed to establish a stable co-culture system using IFRS1 cells and ND7/23 sensory neuron-like cells. **Methods**: ND7/23 cells were seeded at a low density (2 × 10^3^/cm^2^) and maintained for 7 days in serum-containing medium supplemented with NGF (10 ng/mL) and the Rho kinase inhibitor Y27632 (5 μM) to promote neurite elongation. The cells were then treated with the anti-mitotic agent mitomycin C (1 μg/mL) for 12–16 h to suppress proliferative activity. Following this, the cells were co-cultured with IFRS1 cells (2 × 10^4^/cm^2^) and maintained at 37 °C in serum-containing medium supplemented with ascorbic acid (50 μg/mL), NGF (10 ng/mL), and ciliary neurotrophic factor (10 ng/mL). **Results**: Double-immunofluorescence staining performed on day 21 of the co-culture revealed myelin protein 22- or myelin basic protein-immunoreactive IFRS1 cells surrounding βIII tubulin-immunoreactive neurites emerging from ND7/23 cells. Myelin formation was further confirmed via Sudan Black B staining and electron microscopy. **Conclusions**: This co-culture system may provide a valuable tool for studying the processes of myelination in the peripheral nervous system, as well as the pathogenesis of various sensory neuropathies and potential novel therapeutic approaches for these conditions.

## 1. Introduction

As glial cells in the peripheral nervous system (PNS), Schwann cells (SCs) provide trophic support for neuronal growth and maintenance and ensheathe their axons in either a myelinated or an unmyelinated form. Following axonal injury, SCs proliferate, migrate, collaborate with macrophages to clear axon debris, promote the elongation of new axons, and ultimately remyelinate them [1]. Abnormalities in SCs and/or their crosstalk with neurons can lead to demyelinating neuropathy or myelinopathy, and they have also been implicated in neuronopathy and axonopathy [2,3].

Co-culture systems of neurons and SCs have been widely used to investigate their interactions and the molecular and signaling pathways involved in myelination and demyelination [4,5,6,7]. In most previous studies, both neurons and SCs were obtained through primary culture, a time-consuming process requiring extensive preparation to achieve high purity before each co-culture experiment. To improve research efficiency, it would be desirable to establish co-culture systems using cell lines for both neurons and SCs. However, their high proliferative activity and phenotypic differences from primary cultured cells pose significant challenges for achieving stable neuron–SC interactions. Some established SC lines have demonstrated the ability to myelinate neurites in co-culture with primary cultured dorsal root ganglion (DRG) neurons [8,9]. Additionally, a myelinating co-culture system using human-induced pluripotent stem (iPS) cell-derived neurons and neonatal rat SCs has been developed [10]. However, little attention has been given to achieving myelination in co-cultures composed solely of pure neuronal and SC lines.

We previously established a spontaneously immortalized SC line, IFRS1, from long-term primary cultures of adult Fischer344 rat DRG and peripheral nerves [11]. IFRS1 cells retain an intrinsic ability to myelinate neurites in co-culture with primary cultured adult rat DRG neurons [11], nerve growth factor (NGF)-primed PC12 cells [12], mouse embryonic stem (ES) cell line-derived motor neurons [13], and the mouse neuroblastoma/mouse motor neuron hybrid NSC-34 cell line [14,15]. NSC-34 cells were seeded at an approximate density of 2 × 10^3^ cells/cm^2^ and cultured in serum-containing medium supplemented with non-essential amino acids (NEAAs) and brain-derived neurotrophic factor (BDNF) [16,17]. The cells were then pre-treated with the anti-mitotic agent mitomycin C (MMC; 1 μg/mL) for 12–16 h [18] prior to co-culture with IFRS1 cells. This treatment effectively prevented excessive proliferation without inducing cell death. In addition to these models, this experiment aims to establish a myelinating co-culture system using IFRS1 cells and the mouse neuroblastoma/rat embryonic DRG neuron hybrid ND7/23 cell line [19,20]. The co-culture procedure for ND7/23–IFRS1 is similar to that used for NSC-34–IFRS1; however, several distinct differences exist between the two systems, reflecting the biological characteristics of ND7/23 and NSC-34 cells as sensory and motor neuron-like cells, respectively.

## 2. Materials and Methods

All the experiments were conducted at the Tokyo Metropolitan Institute of Medical Science, Tokyo, Japan.

### 2.1. Culture of ND7/23 and IFRS1 Cells

ND7/23 cells were kindly provided by Prof. Atsufumi Kawabata and Dr. Fumiko Sekiguchi of Kindai University, Osaka, Japan, and stored at an approximate density of 1 × 10^5^ cells/mL in Recovery^TM^ cell culture freezing medium (Thermo Fisher Scientific Inc., Waltham, MA, USA) at −80 °C in an ultra-low-temperature freezer (REVCO^TM^, Thermo Fisher). The cells were thawed at 37 °C in a constant-temperature shaking bath (Tokyo Rikakikai Co., Ltd., Tokyo, Japan) and seeded in 100 mm plastic dishes (Greiner Bio-One GmbH, Frickenhausen, Germany) at an approximate density of 1 × 10^4^ cells/cm^2^ at 25 °C in a laminar flow cabinet (Thermo Fisher). Then the cells were maintained in Dulbecco’s Modified Eagle’s Medium (DMEM; Thermo Fisher) supplemented with 5% fetal bovine serum (FBS; Thermo Fisher) and an antibiotic–antimycotic solution (100 U/mL penicillin, 100 μg/mL streptomycin, and 250 ng/mL amphotericin B; Sigma, St. Louis, MO, USA) at 37 °C in a CO_2_ incubator (PHCbi, Tokyo, Japan). Due to the high proliferative activity of ND7/23 cells, we carefully adjusted the seeding density and avoided overgrowth by performing frequent passages (two or three times per week).

IFRS1 cells were established from a long-term primary culture of adult Fischer344 rat SCs in our laboratory [11] and stored at −80 °C, in a similar manner to that of the ND7/23 cells. The cells were thawed at 37 °C, seeded in 100 mm plastic dishes at an approximate density of 2 × 10^4^ cells/cm^2^ at 25 °C, and maintained in DMEM supplemented with 5% FBS, 20 ng/mL recombinant human heregulin-β (EMD Millipore, Billerica, MA, USA), 5 μM forskolin (Sigma), and antibiotic–antimycotic solution at 37 °C. The cell passages were performed once or twice per week.

### 2.2. Co-Culture of ND7/23 and IFRS1 Cells

**Figure 1:**
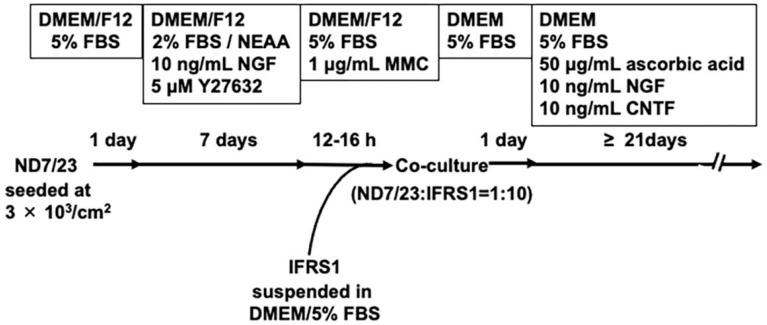
Schematic representation of the entire process of co-culturing ND7/23 and IFRS1 cells.

At semi-confluency, ND7/23 cells were detached using Cell Dissociation Buffer (Thermo Fisher) at 25 °C in a laminar flow cabinet, suspended in Dulbecco’s Modified Eagle’s Medium/Nutrient Mixture F-12 (DMEM/F12; Thermo Fisher) containing 5% FBS in a 50 mL polypropylene conical tube (Greiner Bio-One GmbH). The cells were reseeded onto poly-L-lysine (10 μg/mL; Sigma)-coated Aclar fluorocarbon coverslips (9 mm diameter; Nissin EM Co., Ltd., Tokyo, Japan), type I collagen-coated 12-well culture plates (Corning Inc., Corning, NY, USA), and type I collagen-coated 2-well glass chamber slides (Matsunami Glass Ind., Ltd., Osaka, Japan) at a density of approximately 3 × 10^3^ cells/cm^2^. Cells were maintained in DMEM/F12 supplemented with 2% FBS, 1% NEAAs (FUJIFILM Wako Pure Chemical Corporation, Osaka, Japan) [21], 10 ng/mL recombinant rat NGF (Peprotech, Rocky Hill, NJ, USA), and 5 μM Y27632, a selective Rho-associated coiled coil-forming kinase (ROCK) inhibitor (FUJIFILM Wako) [22], for 5–7 days at 37 °C in a CO_2_ incubator. NGF and Y27632 were added to the culture medium, based on prior studies. ND7/23 cells express tropomyosin receptor kinase A (TrkA), the high-affinity receptor for NGF, and NGF at this concentration is known to promote neurite outgrowth [23]. Y27632 has been reported to enhance neurite outgrowth from primary cultured DRG neurons [22]. When neurite elongation was observed in most ND7/23 cells under phase-contrast microscopy, the cells were incubated for 12–16 h with DMEM/F12 containing 5% FBS and 1 μg/mL MMC (Nacalai Tesque Inc., Kyoto, Japan) [15]. After rinsing twice with DMEM/5% FBS, the cells were co-cultured with IFRS1 cells, which had been detached using 0.05% trypsin/0.53 mM ethylenediaminetetraacetic acid solution (Nacalai Tesque) and suspended in DMEM containing 5% FBS at approximately 1 × 10^5^ cells/mL. The ND7/23–IFRS1 cell ratio was adjusted to approximately 1:10 at 25 °C in a laminar flow cabinet. The co-cultures were fed twice weekly with DMEM containing 5% FBS, 50 μg/mL ascorbic acid (Wako), 10 ng/mL NGF, and 10 ng/mL recombinant rat ciliary neurotrophic factor (CNTF; Peprotech), and they were maintained for longer than 21 days at 37 °C in a CO_2_ incubator (Figure 1). Ascorbic acid functions as both an antioxidant neuroprotective agent [24] and a promotor of myelination by enhancing the synthesis and assembly of extracellular matrix proteins [25]. NGF is essential for the survival of neonatal DRG neurons [26], while CNTF has been shown to support the survival of both DRG neurons [27] and neuroblastoma cells [28].

### 2.3. Immunofluorescence

Double-immunofluorescence staining was performed on day 28 of co-culturing. The cells were fixed with 4% paraformaldehyde for 10 min at 4 °C (in a refrigerator) and permeabilized with 0.1% Triton X-100 in phosphate-buffered saline (PBS) for 5 min at room temperature. Cells were incubated overnight at 4 °C with the following primary antibodies diluted in 20 mM PBS containing 0.4% Block Ace (DS Pharma Biomedical Co., Ltd., Osaka, Japan):

(1) Rabbit anti-myelin basic protein (MBP) polyclonal antibody (1:1000; Merk (formerly Chemicon), Temecula, CA, USA) [11] and mouse anti-βIII tubulin monoclonal antibody (1:1000; Sigma) [29].
(2) Rabbit anti-peripheral myelin protein 22 (PMP22) polyclonal antibody (1:1000, Sigma) [30] and mouse anti-βIII tubulin monoclonal antibody.

After washing with PBS, cells were incubated for 1 h at 37 °C with Alexa Fluor 594 or 488-conjugated anti-rabbit IgG and/or anti-mouse IgG antibody (1:200, Thermo Fisher), followed by nuclear staining with 4’,6-diamidino-2-phenylindole (DAPI, 300 nM; Thermo Fisher) for 5 min at room temperature. Immunocytochemical controls lacking primary antibodies showed no positive staining. The details of antibody validation (Western blotting, pre-absorption tests, etc.) are provided in the cited references. The number of MBP- or PMP22-immunoreactive IFRS1 cells attached to a neurite in each photomicrograph was counted in a similar manner to our previous study [31].

### 2.4. Sudan Black B Staining

Sudan Black B staining was performed on day 28 of co-culturing. Co-cultured cells were fixed on 2-well chamber slides overnight at 4 °C with 4% paraformaldehyde in 0.1 M phosphate buffer (pH 7.4). After post-fixation with 0.1% osmium tetroxide (FUJIFILM Wako) for 1 h at room temperature and dehydration with 70% ethanol, the cells were stained with 0.5% Sudan Black B (FUJIFILM Wako) in 70% ethanol for 1 h at room temperature, rehydrated, and mounted in glycerol gelatin (Sigma) [12].

### 2.5. Image Presentation

The cells were observed at each culture stage (Figure 1) using a cell culture microscope (CKX53; Olympus, Tokyo, Japan). Phase-contrast images were acquired using a microscope digital camera system (DP22-CU; Olympus) with image analysis software (WinROOF2015 Standard; Mitani Corporation, Tokyo, Japan). Cytochemically processed culture samples were observed and imaged using a TCS SP5 confocal microscope system (Leica Microsystems, Wetzlar, Germany).

### 2.6. Electron Microscopy

Electron microscopy was performed on day 28 of co-culturing. Co-cultures in Aclar dishes and 2-well chamber slides were fixed with 3% glutaraldehyde in 0.1 M phosphate buffer for 20 min at room temperature. After washing, cells were postfixed with 1% osmium tetroxide in buffer for 30 min at room temperature, rinsed, dehydrated through graded ethanol, and embedded in epoxy resin. Coverslip portions were removed and re-embedded for cross-sectioning co-cultures [12]. Semi-thin sections (1 μm) were examined using a light microscope (Olympus, Tokyo, Japan), and suitable areas were selected for ultrathin sectioning. Ultrathin sections were double-stained with uranyl acetate and lead citrate, and were examined using an electron microscope (Hitachi, Tokyo, Japan).

### 2.7. Statistical Analysis

No statistical analyses were performed in this study.

## 3. Results

### 3.1. Differentiation of ND7/23 Cells Prior to Co-Culturing

**Figure 2:**
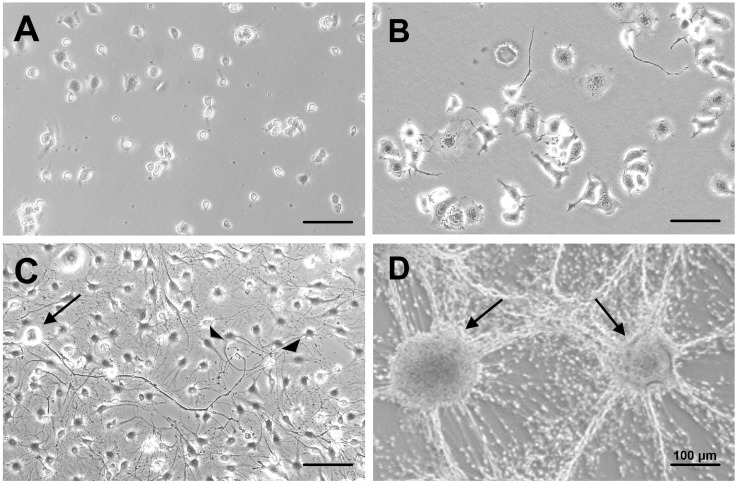
Representative phase-contrast photomicrographs of ND7/23 cells at 1 day (**A**) and 5 days (**B**) of culturing and ND7/23–IFRS1 co-cultures at 2 days (**C**) and 21 days (**D**). At 5 days of culturing, most ND7/23 cells had differentiated from a round morphology (**A**) into flat or polygonal shapes with neurite elongation (**B**). At 2 days of co-culturing, ND7/23 cells were identified by their phase-bright cell bodies (arrow in (**C**)), with neurites extending to the right, while IFRS1 cells exhibited spindle-shaped morphology (arrowheads in (**C**)). At 21 days of co-culture, ND7/23 cells formed aggregates of cell bodies (arrows in (**D**)) from which neurite bundles extended in multiple directions. Some IFRS1 cells adhered to the neurites. The scale bars in (**A**–**D**) are all 100 μm. The experiments were repeated three times with similar results.

We followed a protocol previously shown to suppress proliferation and maintain the viability of NSC-34 cells and other neurons [15,16,17]. During the preliminary trials, we observed that 2% FBS was more effective than 1% in supporting ND7/23 cell survival. After 5–7 days of co-culturing, most cells exhibited morphology differentiated from a rounded shape (Figure 2A) to a flat or polygonal form, with elongated neurites (Figure 2B). The addition of 10 ng/mL NGF and 5 μM Y27632 appeared to exert neurite outgrowth-promoting activities in a similar manner to primary cultured DRG neurons [22,32].

### 3.2. Maintenance of Co-Culture with Stable Neuron–SC Interactions

Consistent with our previous study using NSC-34 cells [15], the pre-treatment of ND7/23 cells with 1 μg/mL MMC for 12–16 h effectively suppressed their excessive proliferation following co-culturing with IFRS1 cells. Co-cultures without MMC pre-treatment resulted in the overgrowth of ND7/23 cells, which hindered the progress of the experiment. To reduce MMC-induced cytotoxicity, the co-cultures were maintained in medium supplemented with 5% FBS, ascorbic acid, NGF, and the neuroprotective molecule CNTF (Figure 2C). These culture conditions enabled the long-term (>21 days) maintenance of co-cultures of MMC-treated ND7/23 cells with IFRS1 cells. After 21–28 days of co-culturing, ND7/23 cells formed cell body aggregates from which neurite bundles extended in multiple directions. Many IFRS1 cells migrated and adhered to the neurites (Figure 2D). These morphological features were similar to those observed in our previous co-culture models [11,12,15], indicating stable and effective axon–SC interactions as a prerequisite for successful myelination.

### 3.3. Myelination in NSC-34–IFRS1 Co-Cultures

**Figure 3:**
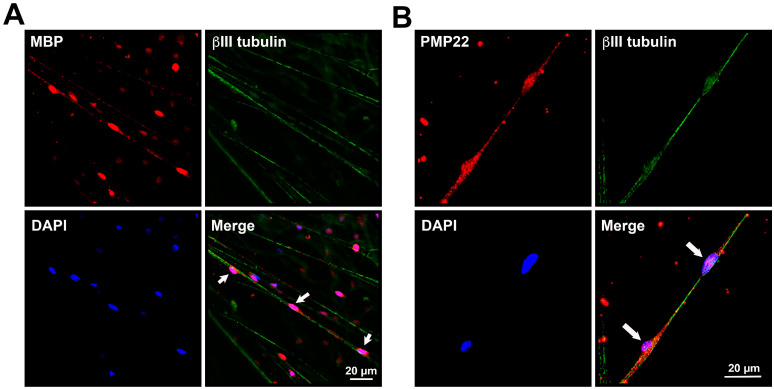
Representative triple-immunofluorescence photomicrographs of ND7/23-IFRS1 co-cultures at 28 days. Myelin formation was demonstrated by MBP (**A**)—or PMP22 (**B**)—positive IFRS1 cells (red) surrounding βIII tubulin-positive neurites (green). Nuclei were stained blue with DAPI. Myelin-like segmental structures are indicated as arrows in the Merge pictures. The experiments were repeated three times with similar results.

**Figure 4:**
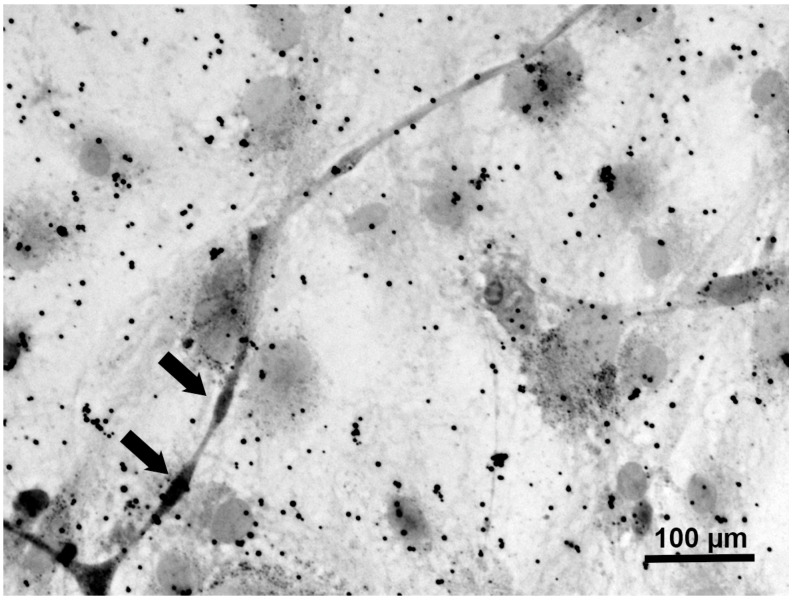
A representative photomicrograph of ND7/23-IFRS1 cells at 28 days of co-culturing stained with Sudan Black B. Myelin segments are indicated as arrows. The experiments were repeated three times with similar results.

**Figure 5:**
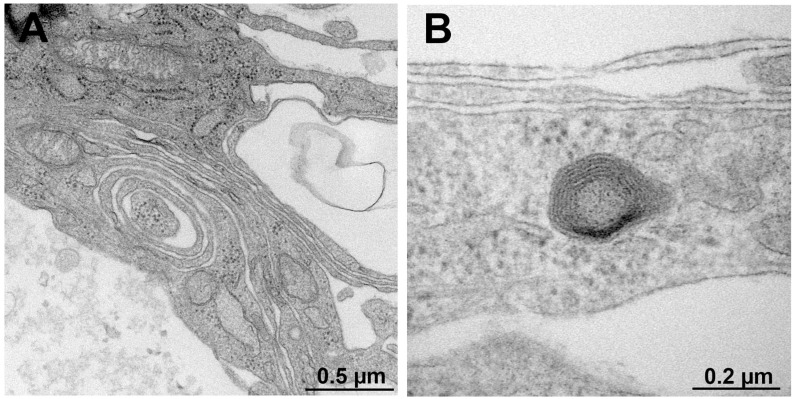
Representative electron micrograph of transversely sectioned promyelinating (**A**) and myelinating (**B**) structures in ND7/23-IFRS1 cells at 28 days of co-culturing. The experiments were repeated three times with similar results.

After 28 days of co-culturing, myelin formation was verified via light and electron microscopy. As the co-cultures at this stage were fragile, with an increased cell-free area among neurite bundles (Figure 2D), we made sure to prevent the cells from detaching from the dishes. Double-immunofluorescence staining revealed that IFRS1 cells expressing myelin protein (MBP or PMP22) surrounded βIII tubulin-positive neurites (Figure 3). The average number of MBP- or PMP22-positive IFRS1 cells attached to a neurite is 2.47 ± 0.83 (mean ± SD, N = 50). Additionally, myelin segments were visualized by Sudan Black B staining (Figure 4). These light microscopic findings were consistent with the formation of promyelinating and myelinating structures, as confirmed by electron microscopy (Figure 5). These results demonstrate that the current protocol enables IFRS1 cells to successfully myelinate neurites extending from ND7/23 cells.

## 4. Discussion

This experiment establishes a myelinating co-culture system using ND7/23 sensory neuron-like cells and IFRS1 Schwann cells. IFRS1 cells have been shown to myelinate neurites in co-culture not only with primary cultured DRG neurons [11] but also with neuronal cell lines such as NGF-primed PC12 cells [12] and NSC-34 motor neuron-like cells [15]. The strategies employed to suppress cell proliferation, promote neurite outgrowth, and maintain the long-term survival of ND7/23 cells were fundamentally similar to those used in the NSC-34–IFRS1 co-culture system [15] but were modified to accommodate the phenotypic differences between ND7/23 and NSC-34 cells (Figure 1). Notably, TrkA is expressed in ND7/23 cells [25] but not in NSC-34 cells [33]. NGF, known to be essential for the survival of immature DRG neurons [29,34], also exerts neurotrophic effects on ND7/23 cells [23]. Therefore, ND7/23 cells were maintained in the presence of NGF prior to co-culturing, while BDNF was used for NSC-34 cells due to its neurotrophic effects on these TrkB (the high-affinity BDNF receptor)-expressing cells [17]. During co-culturing with IFRS1 cells, NGF was continuously supplied to support neurite elongation and maintenance in ND7/23 cells. Additionally, since RhoA and its downstream target ROCK are well-known inhibitors of axonal regeneration [35] and RhoA inhibition promotes neurite outgrowth in ND7/23 cells [26], we used Y-27632 instead of RhoA inhibitors. Y27632 has been reported to promote neurite outgrowth in both primary cultured DRG neurons and neuronally differentiated PC12 cells [22,36].

The primary obstacle in maintaining the co-culture was the high proliferative activity of ND7/23 cells. Although most ND7/23 cells exhibited morphological differentiation with neurite elongation in response to NGF and Y27632 (Figure 2B,C), excessive proliferation remained unavoidable when directly co-cultured with IFRS1 cells. This may be attributed to the neuroblastoma components of ND7/23 cells [19,20], as well as the secretion of various growth-stimulating factors by IFRS1 cells [11]. In our previous study [15], pre-treatment with MMC (1 μg/mL for 12–16 h) effectively suppressed the overgrowth of NSC-34 cells in co-culture with IFRS1 cells. In the present study, the same strategy was successfully applied to ND7/23 cells. To mitigate MMC-induced cytotoxicity, the co-cultures were maintained in serum-containing medium supplemented with neuroprotective agents such as ascorbic acid [24], NGF [26], and CNTF [27,28]. The neuroprotective effects of CNTF are likely mediated through the activation of the Janus kinase 2 (JAK2)/signal transducer and activator of transcription 3 (STAT3) and phosphatidyl inositol-3’-phosphate-kinase (PI3K)/serine-threonine protein kinase (AKT) pathways [27], as well as the enhancement of mitochondrial bioenergetics via nuclear factor-kappa B (NF-κB) activation [37]. Although some ND7/23 cells underwent degeneration and death largely due to the cytotoxic effects of MMC, this did not interfere with the sustained interactions between axons and SCs. Supporting this finding, the intra-striatal injection of murine embryonic stem cells pre-treated with 1 μg/mL MMC for 12 h ameliorated motor dysfunction in a murine model of Parkinson’s disease without inducing tumor formation or neuronal cell death for up to 15 months [18]. These findings suggest that MMC pre-treatment does not compromise long-term neuronal health. After 21–28 days of co-culturing, IFRS1 cells were observed to migrate and adhere to the neurite networks extending from ND7/23 cell body aggregates (Figure 2D). These morphological changes suggested successful myelination, which was confirmed by immunofluorescence (Figure 3), Sudan Black B staining (Figure 4), and electron microscopy (Figure 5). Light microscopy clearly demonstrated segmental myelin structures (Figure 3 and Figure 4), while electron micrographs revealed transverse views of promyelinating (Figure 5A) and small myelinating structures (Figure 5B), although typical compact myelin sheaths, as observed in the co-cultures with IFRS1 cells and primary cultured DRG neurons [38], were not present. This discrepancy may be due to the greater difficulty in forming compact myelin sheaths when using neuronal cell lines compared to primary cultured neurons. A longer duration (>4 weeks) of ND7/23⎼IFRS1 co-culturing may be required for the development and maturation of myelin sheath; however, most co-cultures became fragile after 5 weeks and detached from the dishes by 6 weeks. Optimizing the methodology to support long-term co-culturing remains an important subject for future research. Such longer duration of co-culture will enable us to observe the demyelination process due to MMC-induced neurodegeneration. In our previous co-culture model using PC12 and IFRS1 cells [12], electron micrographs showed incomplete compact myelin structures, such as myelin sheaths surrounding axons and cell-free areas. The lack of typical compact myelin may be attributed to the use of neuronal cell lines, although the precise reason remains unclear. Nevertheless, amiodarone-induced Schwann cell injury and demyelinating neuropathies were successfully demonstrated by biochemical and light microscopic analyses in the PC12-IFRS1 co-culture model [31]. Therefore, we believe that the ND7/23-IFRS1 co-culture model will also be applicable for studying mature myelin. The number of myelin structures identified by light microscopy in the co-cultures was approximately 2.5/neurite, insufficient to evaluate the percentages of myelinated neurites, myelin thickness, or segment length. Furthermore, the longevity of this culture system is limited to less than 6 weeks, which may hinder its application in the study of chronic demyelinating diseases such as chronic inflammatory demyelinating polyneuropathy (CIDP). However, this model remains suitable for investigating acute demyelinating neuropathies, including Guillain–Barré syndrome [39] and drug-induced demyelinating conditions [40]. We plan to investigate the effects of anti-ganglioside antibodies and dichloroacetate in the ND7/23-IFRS1 co-culture [31].

Although we have not performed a direct comparison, we can safely state that IFRS1 cells myelinate primary cultured DRG neurons more efficiently than ND7/23 cells. Since primary cultured DRG neurons do not proliferate during co-culturing with IFRS1 cells, MMC pre-treatment and subsequent detoxification steps are unnecessary. Additionally, transcriptomic analyses have revealed that the gene expression profile of ND7/23 is somewhat distinct from that of whole DRG tissue [41]. This distinction may contribute to the reduced myelination efficiency of ND7/23 cells compared to primary cultured DRG neurons, although further investigation is required.

Another limitation of this study is that we only presented morphological findings without electrophysiological or functional assays. Although such functional analyses would undoubtedly strengthen the study, they are considerably more challenging to conduct than morphological analyses. Our ongoing research focuses on improving the co-culture method to achieve more efficient myelination, which we consider a prerequisite for further functional studies. Additionally, as this was a nonrandomized, unblinded study, we plan to conduct a follow-up study with the quantitative analyses evaluating the effects of NEAAs, NGF, and CNTF concentrations on the number of myelin-like structures.

Recent studies have demonstrated myelination in co-culture systems using iPS cell-derived neurons and SCs [42], and some of these cell types are now commercially available. However, the purchase of iPS-derived cells is costly, and practical, standardized protocols for achieving myelination in such co-cultures have yet to be fully established. In contrast, our co-culture models using IFRS1 cells with NGF-primed PC12 cells [12], NSC-34 cells [15], and ND7/23 cells (this study) consist of well-characterized neuronal and SC lines that can be prepared and maintained using straightforward culture techniques, without the need for genetic manipulation. The NSC-34–IFRS1 co-culture model may be particularly suited for studies of motor neuron diseases, while the ND7/23⎼IFRS1 co-culture model may provide a useful platform for investigating diabetic and other sensory neuropathies. Future research should determine whether high-glucose conditions [43] or advanced glycation end products (AGEs) and their precursors (e.g., glycolaldehyde [44]) induce axonal degeneration and demyelination-like changes in co-culture systems.

## Data Availability

All data presented in this paper are available in the manuscript or from the corresponding author (K.S.).

## Abbreviations

The following abbreviations are used in this manuscript:

AGEs	Advanced Glycation End Products
BDNF	Brain-Derived Neurotrophic Factor
CNTF	Ciliary Neurotrophic Factor
DMEM	Dulbecco’s Modified Eagle’s Medium
DMEM/F12	Dulbecco’s Modified Eagle’s Medium/Nutrient Mixture F-12
FBS	Fetal Bovine Serum
IFRS1	Immortalized Fischer344 Rat Schwann Cells 1
iPS	Induced Pluripotent Stem (Cells)
JAK2	Janus Kinase 2
MBP	Myelin Basic Protein
MMC	Mitomycin C
NEAA	Non-Essential Amino Acids
NF-κB	Nuclear Factor-Kappa B
NGF	Nerve Growth Factor
PI3K	Phosphatidyl Inositol-3’-Phosphate-Kinase
PMP22	Peripheral Myelin Protein 22
PNS	Peripheral Nervous System
ROCK	Rho-associated Coiled-Coil Forming Kinase
SCs	Schwann Cells
STAT3	Signal Transducer and Activator of Transcription 3
